# Psychosocial stress from pre- to post-pandemic times: latent class mixed model analysis using data from a German cohort

**DOI:** 10.1007/s00127-026-03092-6

**Published:** 2026-04-20

**Authors:** Daniela Costa, Johannes Horn, Janka Massag, Oliver Purschke, Alexander Kluttig, Amand Führer, Rafael Mikolajczyk

**Affiliations:** Institute of Medical Epidemiology, Biometrics, and Informatics, Medical Faculty of the Martin Luther University Halle-Wittenberg; Magdeburger Str. 8, 06112 Halle (Saale), Germany

**Keywords:** Covid-19, Growth mixture models, Mental health, Psychological stress, Trajectories

## Abstract

**Purpose:**

The COVID-19 pandemic and containment measures disrupted daily life and worsened mental health. Stress, a key driver of mental disorders, likely intensified during this period. However, longitudinal studies tracking stress trajectories in the general population remain limited. This study aims to identify psychosocial stress trajectories from the pre- to post-pandemic period and examine associated characteristics in a population-based sample from a German city.

**Methods:**

966 participants from the German National Cohort study-centre in Halle (240,000 inhabitants in Eastern Germany) were included. Those participated in a six-monthly intensified assessment and completed at least four questionnaires between 2019 and 2024 containing the PHQ-Stress module. First, latent class mixed models analysis identified heterogeneous stress trajectories. Second, associations between the most likely class membership and covariates were tested with multinomial and binomial logistic regressions.

**Results:**

We identified four psychosocial stress trajectory classes. Most participants followed a resilient trajectory (82%), while others showed chronic (4%), recovered (5%), or delayed (9%) trajectories. Membership in the resilient trajectory was associated with lower pre-pandemic psychological vulnerability, higher life satisfaction, greater agreeableness, and older age.

**Conclusion:**

Resilience predominated, while smaller subgroups showed less adaptive trajectories, with prior stress levels shaping long-term patterns. These findings demonstrate heterogeneity in stress responses and support the applicability of resilience frameworks such as Bonanno’s.

**Supplementary Information:**

The online version contains supplementary material available at https://doi.org/10.1007/s00127-026-03092-6.

## Background

Mental disorders have a multifactorial origin, emerging from a complex interplay of biological, psychological, and social factors [1–4], such as early life trauma [5], social isolation [6], academic and work pressure [7, 8], economic instability [8, 9], socio-political dynamics [10], and major global events [11–13]. Psychosocial stress is a common pathway linking these factors and is relevant to the development and exacerbation of mental disorders [14, 15]; chronic stress can also impair physical health [16]. Thus, stress is an important target for prevention [17].

The COVID-19 pandemic and subsequent containment measures profoundly disrupted daily life and increased uncertainty [18–20], becoming a combination of multifaceted stressors and potentially exacerbating pre-existing strains [21]. According to the transactional stress model, stress responses depend on primary appraisal (evaluation of demands) and secondary appraisal (perceived coping resources) [22], indicating that a shared stressor does not necessarily leads to homogeneous responses. Early studies, mainly cross-sectional and based on convenience samples [23, 24], suggested widespread elevated distress symptoms [25, 26], with many focusing on considered high-risk groups, such as individuals with comorbidities or pre-existing health conditions [27], and pandemic-related essential workers [28], rather than population-based samples.

By late 2020, systematic reviews began reporting heterogeneity in mental health responses, questioning the hypothesis of a general deterioration, and highlighting the need for longitudinal assessments containing pre-pandemic data [26, 29]. Longitudinal studies with pre-pandemic data reported an average deterioration in mental health in early 2020, followed by a general attenuation of symptoms, although the magnitude of change varied across populations [30–32]. Importantly, population-average changes may obscure distinct stress trajectories from small subgroups. Evidence from research on major adversities shows that most individuals follow resilient trajectories, while experiencing chronic or reactive distress is less common [33, 34]. Studies considering heterogeneous mental health reactions to the pandemic have reported that younger adults, women, from lower socioeconomic status, and those with pre-existing mental health conditions experienced greater distress [32, 35, 36].

Despite growing acknowledgement of the importance of investigating the longitudinal changes and heterogeneity, greater attention has been given to depression and anxiety symptoms [37]. Studies from Germany investigating trajectories of stress during early pandemic phases, provided some insights, identifying 3 to 4 heterogeneous trajectories, where resilience predominated [38, 39]. However, longitudinal research on psychosocial stress that includes pre-pandemic data and extends beyond the first weeks or months, and relies on population-based samples remains scarce. Addressing these limitations is important to better characterise heterogeneity in stress responses and to inform preparedness for future macro-stressors.

With this background, this study aims [1] to identify psychosocial stress trajectories among residents from an urban population and its surrounding rural areas in Germany, covering the period from before the COVID-19 pandemic onset until a late stage; and [2] examine how trajectory membership is associated with participants’ characteristics from three specific data-collection points: baseline (three to five years before pandemic), shortly before the pandemic, and early in the pandemic.

## Methods

### Participants

This study is part of the German National Cohort (NAKO Gesundheitsstudie), a population-based study conducted at 18 study centres across Germany. Study design details have been published elsewhere [40]. In brief, between 2014 and 2019, approximately 205,000 individuals were recruited and examined. The first follow-up examination (FU1) was conducted about five years after enrollment (2019–2024). Data was collected via medical exams, face-to-face interviews, and touch-screen questionnaires. During the COVID-19 pandemic, two additional questionnaires were administered, in May 2020 and September 2022, distributed by e-mail or regular mail.

In addition to the general study examinations, supplemental modules were implemented at selected NAKO centres as Level-3 projects. At the Halle (Saale) centre, we initiated an intensified six-month follow-up in 2019 for participants who agreed to receive additional online surveys, on physical activity, stress, and sleep.

**Fig. 1:**
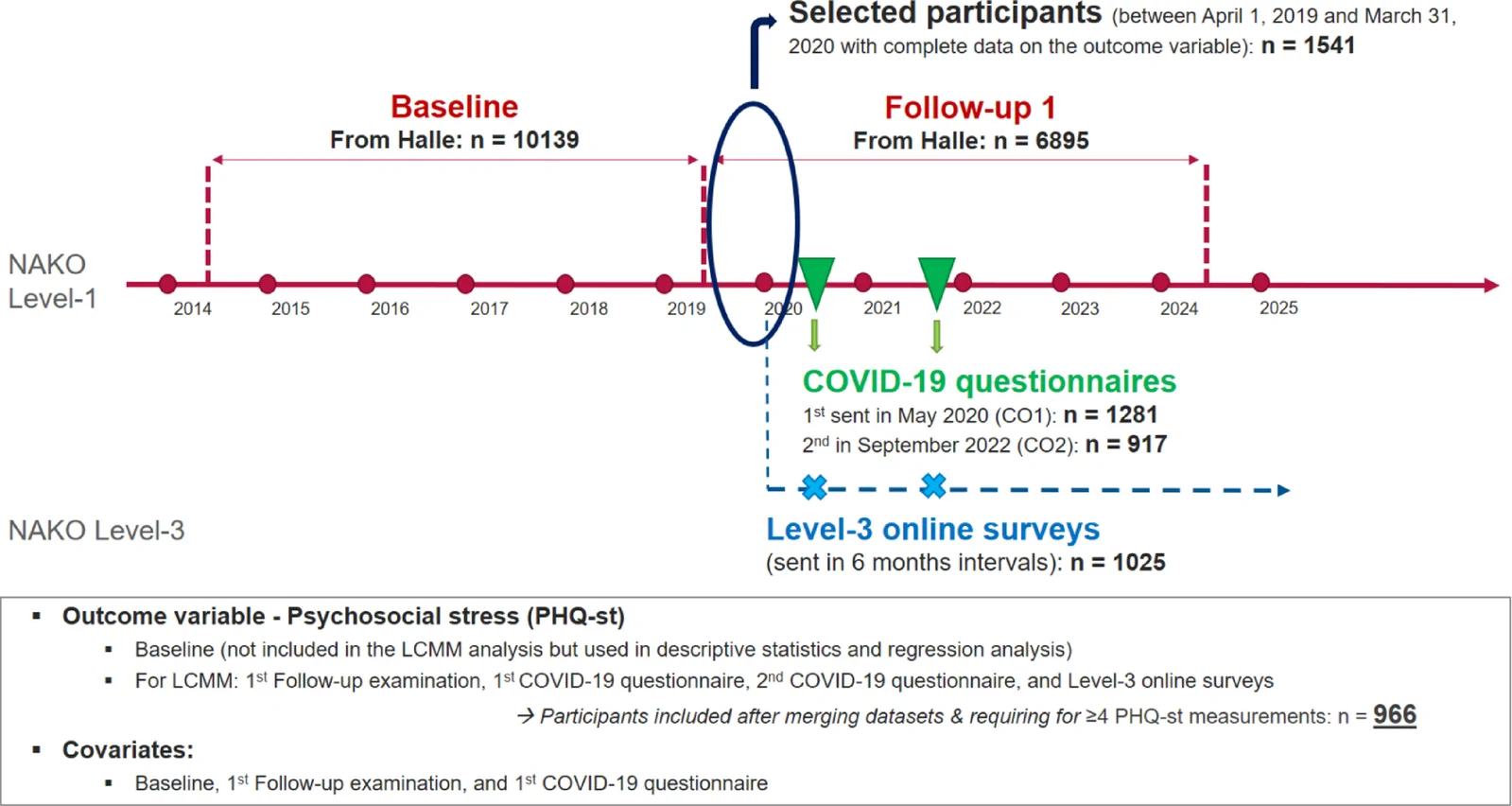
Timeline of NAKO study, the data collection used in the current study, and participant selection across the different assessments. At Level-1, all the 18 study centres participate, whereas Level-3 is study-centre specific

At baseline, 10,139 individuals were examined in Halle, with 6,895 participating in FU1. For the current study, we included individuals who completed FU1 between April 1, 2019 and March 31, 2020, i.e. before the first COVID-19 containment measures, ensuring a recent pre-pandemic assessment. We aimed to include six-monthly questionnaire data for each participant until their last available assessment, with data collection until early 2024. Participants were included if they had completed at least four questionnaires (Fig. 1).

### Measurements

#### Outcome variable

We assessed psychosocial stress using the German version of the Patient Health Questionnaire – stress module (PHQ-st) [41]. PHQ-st inquires about the previous four weeks and contains ten items. Items are rated on a scale, where 0 means ‘not bothered’, 1 ‘bothered a little’ and 2 ‘bothered a lot’, leading to a score ranging from 0 to 20, where a higher score indicates greater stress (Fig. 1 – Supplementary material (SM)). This total score was used as a continuous variable in all analyses. PHQ-st has good psychometric properties, such as reliability (ω = 0.776) and validity [42].

#### Covariates

To characterise individuals with different stress trajectories, we examined baseline, FU1, and the first COVID-9 questionnaire (CO1) covariates, identified in previous literature as potential predictors of stress and related mental health outcomes. Time-invariant variables were collected at baseline: sex, month and year of birth, personality traits (Big Five) [43], and early childhood trauma (Childhood Trauma Screener) [44].

Potentially time-varying covariates included sociodemographic characteristics (marital and relationship status, education [45]), international social-economic index of occupation status (ISEI) [46, 47], anxiety and depressive symptoms (assessed using GAD-7 and PHQ-9, respectively) [48, 49], health-related quality of life (HRQOL) [50], prior mental disorder diagnoses, body mass index (BMI) [51], and life satisfaction [52, 53]. In addition, we used current employment and housing situation in the beginning of the pandemic, as well as newly introduced items on behavioural changes (physical activity), household financial strain, loneliness [54], and number of fears from CO1. Figure 1 shows the study timeline, data collection and participant selection; further information in SM.

### Statistical analysis

We performed a latent class mixed model (LCMM) analyses, also referred to as growth mixture models, to identify heterogeneous trajectories of psychosocial stress from April 2019 to the beginning of 2024. Analyses were conducted in R 4.4.0 [55], using the lcmm R-package [56]. PHQ-st scores exhibited right-skewed distribution typical of stress measures, and thus it was normalised to adequate use of the hlme function. Time was treated as a continuous variable, with a common time-zero for all participants, and individual time points based on questionnaire completion date. The fixed effects were specified for linear, quadratic, and cubic terms of time. To account for individual variability in trajectories, random effects were specified up to cubic terms of time. The subject identifier was included as a grouping variable to account for the repeated measures. The model including fixed effects up to cubic terms of time and random effects up to the quadratic terms showed the best performance based on fit indices (log-likelihood, Akaike Information Criterion (AIC), and Bayesian Information Criterion (BIC)) and was thus selected for further analyses. All LCMMs were estimate using maximum likelihood. To reduce the risk of convergence to local maxima, a grid search was run up to 30 iterations from 100 random vectors of initial values. The model with the highest log-likelihood was taken as initial values for the final estimation.

**Fig. 2:**
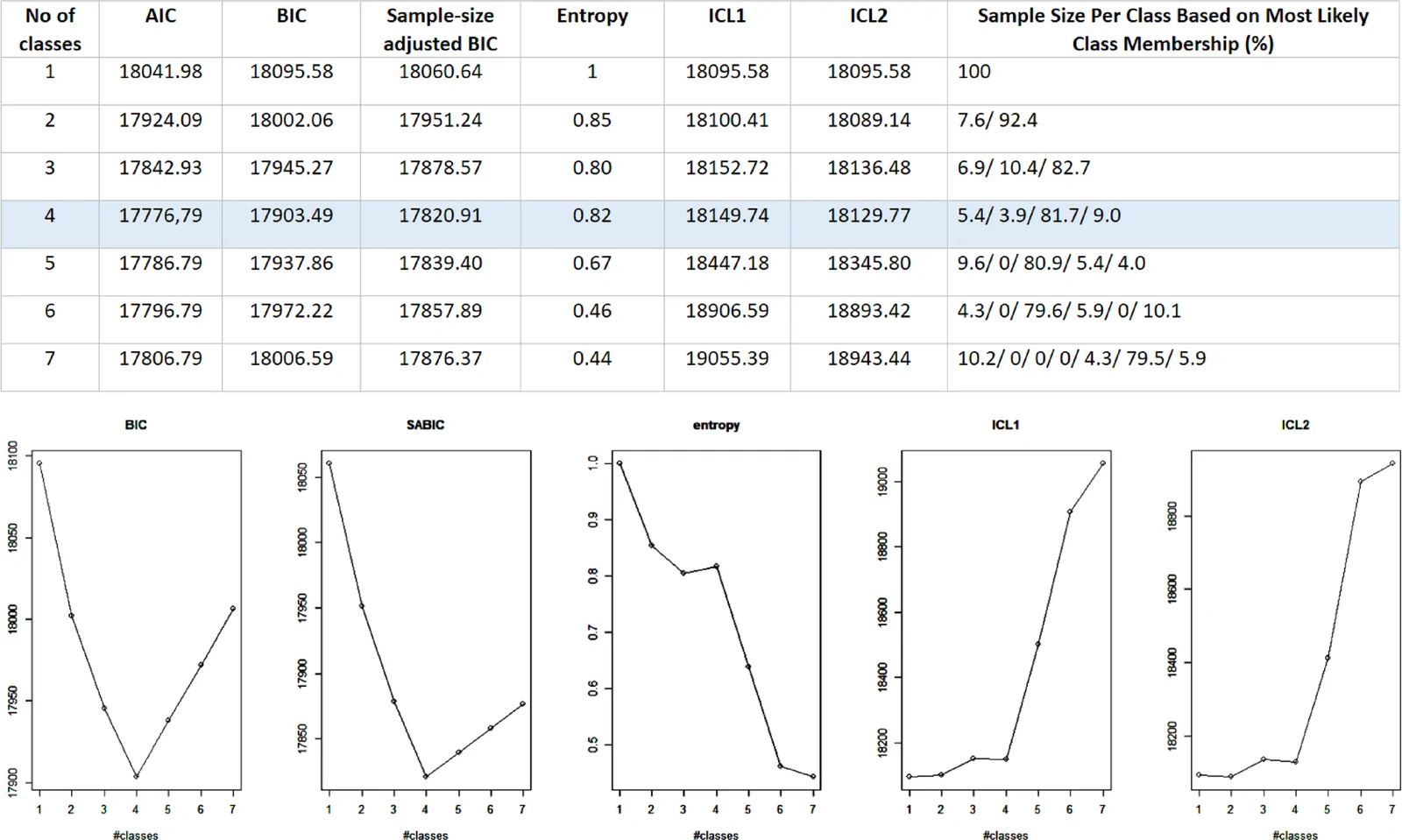
Fit indices from the LCMM analysis, with fixed effects up to cubic terms and random effects up to quadratic terms

We expected the most plausible model to contain four or five classes, this expectation was theoretically informed by Bonanno’s resilience trajectory framework [21, 33] and research on early pandemic trajectories with mental health constructs [17]. Following the approach described by Schoot et al. [57], we progressively fitted models with one to seven classes. Model fit statistics for 1–7 class solutions are presented in Fig. 2. The four-class solution demonstrated the best statistical measures (BIC, sample-size adjusted BIC (SABIC), entropy, and Integrated Completed Likelihood (ICL)). A Bootstrap Likelihood Ratio Test (BLRT) indicated a significant improvement in model fit from 3 to 4 classes (*p* = 0.004; in SM – Fig. 2). Moreover, considering interpretability, conceptual relevance, and practical meaning in elucidating heterogeneous trajectories of psychosocial stress over time, this model also offers the most fitting solution. For each class, a spaghetti plot was created using the ggplot2 R-package [58], to visualize individual trajectories over time, with a LOESS-smoothed trend line capturing the overall trend across individual trajectories.

Following the approach described by Schoot et al. [57], no covariates were included in the LCMM. Instead, the covariates of the class membership were assessed in a subsequent multinomial logistic regression. Overall the percentage of missing data was low, varying across covariates (maximum 7% - SM Table 1). To avoid loss of participants due to complete-case requirements in multinomial regression analysis, missing data were handled using multiple imputation with the mice R-package [59], and performed under the assumption of missing at random (MAR) [60]. Predictive mean matching was used for numeric and ordinal variables. For nominal variables, appropriate regression-based imputation models were specified. Imputation was performed using a dataset containing data from baseline, FU1, and CO1 (6,823 participants), including all variables used in the analysis, as well as a wide range of additional auxiliary variables related to missingness.

Initial covariate selection for the multinomial logistic regression was guided by literature and an examination of a correlation matrix. Additionally, we tested models including variables not previously assessed, but considered relevant in the context of the pandemic. To address small subgroup sizes and improve interpretability, categorical variables were dichotomised where appropriate. Three models were run: The first included only baseline covariates; the second added FU1 time-varying measures (the most recent pre-pandemic data) to the baseline time-invariant variables; and the third additionally incorporated the most recent time-varying data up to early in the pandemic (May 2020). In models two and three only the most recent data were used, ensuring that repeated measurements of the same variable were not simultaneously included in a model. Aiming to restrict predictors to the most essential, models were rerun applying stepwise selection with the MASS R-package [61]. However, this approach did not improve model fit and was therefore not retained. Variance inflation factor values indicated no problematic multicollinearity. Nagelkerke’s R-squared was used as an indicator of the explanatory power of the models and for model comparisons. A binomial logistic regression was also performed using the same covariates as the multinomial logistic models to provide a more stable comparison given the small size of some trajectory classes.

## Results

### Participants’ characteristics

We included 966 participants in the LCMM analysis, who had completed in average 8.5 (SD 1.8) surveys containing the outcome variable between April 2019 and January 2024, with an average of intra-individual interval between assessments of 5.9 (SD 1.8) months. The final analyses included 961 participants, after excluding five participants due to missing Level-3 identification in the baseline dataset.

The mean age of participants in January 2019 was 52.4 years (range 24–74), and 53% (512) of the sample were women. Most participants were married at FU1 (570, 59%), employed at CO1 (689, 72%) and most (861, 90%) reported easy or reasonably easy household financial situation. In FU1, 144 participants (15%) reported a lifetime diagnosis of depression, and 89 (9%) reported a lifetime diagnosis of anxiety and/or panic attacks (Table 1 and 2).

**Table 1:** Participant Characteristics at Baseline, FU1, and CO1, by PHQ-Stress Trajectory Class (Frequencies and Percentages for Categorical Variables)

Collection		All sample, n (%)	PHQ-st trajectories’ classes			
			Recovered, n (%)	Chronic, n (%)	Resilient, n (%)	Delayed, n (%)
	Expected number of participants	961 (100)	52 (5.4)	38 (3.9)	789 (81.6)	87 (9.0%)
Baseline	Sex					
	Female, n (%)	512 (53.3)	33 (63.5)	26 (68.4)	405 (51.7)	48 (55.2)
	Male, n (%)	449 (46.7)	19 (36.5)	12 (31.6)	379 (48.3)	39 (44.8)
	Age in January 2019 (years) - calculated from month and year of birth collected at baseline					
	20-29, n (%)	46 (4.8)	3 (0.3)	2 (0.2)	35 (3.6)	6 (0.6)
	30-39, n (%)	128 (13.3)	13 (1.4)	8 (0.8)	90 (9.4)	17 (1.8)
	40-49, n (%)	198 (20.6)	15 (1.6)	9 (0.9)	159 (16.5)	15 (1.6)
	50-59, n (%)	280 (29.1)	16 (1.7)	15 (1.6)	216 (22.5)	33 (3.4)
	60-69, n (%)	227 (23.6)	4 (0.4)	4 (0.4)	206 (21.4)	13 (1.4)
	70-79, n (%)	82 (8.5)	1 (0.1)	0 (0.0)	78 (8.1)	3 (0.3)
First follow-up examination	Marital status					
	Married, n (%)	570 (59.3)	28 (53.8)	21 (55.3)	481 (61.4)	40 (46.0)
	Married, living apart from spouse, n (%)	17 (1.8)	1 (1.9)	2 (5.3)	14 (1.8)	0 (0)
	Single, n (%)	221 (23.0)	17 (32.7)	11 (28.9)	168 (21.4)	25 (28.7)
	Divorced, n (%)	112 (11.7)	6 (11.5)	3 (7.9)	85 (10.8)	18 (20.7)
	Widowed, n (%)	41 (4.3)	0 (0)	1 (2.6)	36 (4.6)	4 (4.6)
	Ever diagnosed with Depression					
	no, n (%)	817 (85.0)	39 (75.0)	24 (63.2)	694 (88.5)	60 (69.0)
	yes, n (%)	144 (15.0)	13 (25.0)	14 (36.8)	90 (11.5)	27 (31.0)
	Ever diagnosed with Anxiety/ Panic attack					
	no, n (%)	872 (90.7)	45 (86.5)	30 (78.9)	722 (92.1)	75 (86.2)
	yes, n (%)	89 (9.3)	7 (13.5)	8 (21.1)	62 (7.9)	12 (13.8)
	Body Mass Index					
	under-/normal-weight (<25kg/m^2^), n (%)	417 (43.4)	21 (40.4)	12 (31.6)	348 (44.4)	36 (41.4)
	overweight/obesity (≥25kg/m^2^), n (%)	544 (56.6)	31 (59.6)	26 (68.4)	436 (55.6)	51 (58.6)
COVID-19 first questionnaire	Employment status^(5 missing values)^					
	Employed, n (%)	689 (72.1)	46 (88.5)	31 (83.8)	542 (69.5)	70 (80.5)
	Unemployed or Non-working person, n (%)	267 (27.9)	6 (11.5)	6 (16.2)	238 (30.5)	17 (19.5)
	Household Financial Situation ^(4 missing values)^					
	Easy, n (%)	562 (58.8)	25 (48.1)	10 (27)	486 (62.2)	41 (47.1)
	Reasonably easy, n (%)	299 (31.3)	18 (34.6)	15 (40.5)	229 (29.3)	37 (42.5)
	With some difficulties, n (%)	8 (0.8)	1 (1.9)	3 (8.1)	4 (0.5)	0 (0)
	With great difficulty, n (%)	88 (9.2)	8 (15.4)	9 (24.3)	62 (7.9)	9 (10.3)
	Living situation ^(4 missing values)^					
	People living alone, n (%)	176 (18.4)	10 (19.2)	6 (16.2)	140 (17.9)	20 (23)
	Couples without children in the household, n (%)	468 (49.0)	19 (36.5)	13 (35.1)	400 (51.2)	36 (41.4)
	Couples with minor child(ren) in the household, n (%)	201 (21.0)	17 (32.7)	8 (21.6)	159 (20.4)	17 (19.5)
	Couples with adult child(ren) in the household, n (%)	50 (5.2)	2 (3.8)	4 (10.8)	39 (5)	5 (5.7)
	Single parents, n (%)	28 (2.9)	4 (7.7)	2 (5.4)	18 (2.3)	4 (4.6)
	Other multi-person households, n (%)	34 (3.6)	0 (0)	4 (10.8)	25 (3.2)	5 (5.7)

**Table 2:** Mean and Standard Deviation of Participant Characteristics at Baseline, FU1, and CO1, by PHQ-Stress Trajectory Class (Numeric Covariates)

Collection time	Participants	PHQ-st trajectories’ classes				
		All sample, *n* (%)	Recovered, *n* (%)	Chronic, *n* (%)	Resilient, *n* (%)	Delayed, *n* (%)
		961 (100)	52 (5.4%)	38 (3.9%)	789 (81.6%)	87 (9.0%)
	Covariates					
Baseline	BIG 5 Agreeableness score, mean (SD)	5.64 (0.95)	5.28 (0.92)	5.21 (1.06)	5.71 (0.93)	5.44 (1.03)
	BIG 5 Consciousness score, mean (SD)	5.86 (0.91)	5.98 (0.84)	5.76 (1.05)	5.87 (0.89)	5.78 (1.04)
	BIG 5 Extraversion score, mean (SD)	4.71 (1.29)	4.63 (1.25)	4.62 (1.41)	4.71 (1.28)	4.77 (1.39)
	BIG 5 Neuroticism score, mean (SD)	3.50 (1.34)	4.28 (1.42)	4.77 (1.18)	3.36 (1.29)	3.74 (1.33)
	BIG 5 Openness score, mean (SD)	4.69 (1.26)	4.77 (1.23)	4.89 (1.23)	4.68 (1.27)	4.60 (1.27)
	Childhood trauma (CTS score), mean (SD)	6.76 (2.26)	7.46 (2.90)	7.71 (3.09)	6.66 (2.15)	6.79 (2.24)
	Psychosocial stress (PHQ-st), mean (SD)	3.49 (2.97)	5.10 (3.90)	8.63 (4.30)	2.99 (2.40)	4.80 (3.37)
	International socio-economic index (ISEI), mean (SD)	53.08 (15.05)	51.46 (15.35)	48.53 (14.68)	53.52 (15.01)	52.10 (15.21)
FU1	Health-related quality of life (SF36–1 item), mean (SD)	2.69 (0.66)	2.75 (0.84)	3.18 (0.73)	2.65 (0.63)	2.78 (0.72)
	Life satisfaction (L-1), mean (SD)	9.03 (1.66)	8.06 (2.20)	7.53 (2.14)	9.24 (1.49)	8.36 (1.80)
	Anxiety symptoms (GAD-7), mean (SD)	2.91 (2.94)	4.54 (3.30)	8.74 (4.32)	2.38 (2.33)	4.13 (3.45)
	Depressive symptoms (PHQ-9), mean (SD)	3.51 (3.14)	4.98 (3.78)	9.50 (4.60)	2.97 (2.50)	4.92 (3.72)
	Education years (ISCED97), mean (SD)	16.25 (2.07)	15.90 (2.09)	16.11 (1.87)	16.30 (2.07)	16.16 (2.07)
CO1	Loneliness (3-item scale), mean (SD)	4.76 (1.48)	5.38 (1.68)	5.63 (1.79)	4.65 (1.43)	4.97 (1.47)
	Number of fears^a^, mean (SD)	2.55 (2.35)	3.44 (2.22)	4.18 (2.73)	2.40 (2.30)	2.63 (2.30)

Scores ranges:

- Ascending order: Big-5 traits: 1 to 7; CTS: 5 to 25; ISEI: 10 to 89; GAD-7: 0 to 21; number of fears: 0 to 8; L-1: 1 to 11; Loneliness: 3 to 9; PHQ-9: 0 to 27; PHQ-st: 0 to 20;

- Descending order: SF36-1 item: 1 (excellent) to 5 (poor)

^a^ Number of fears among: road traffic accident, natural disaster, COVID-9 infection, cancer, stroke, heart attack, diabetes mellitus, hereditary disease

### PHQ-st trajectories

**Fig. 3:**
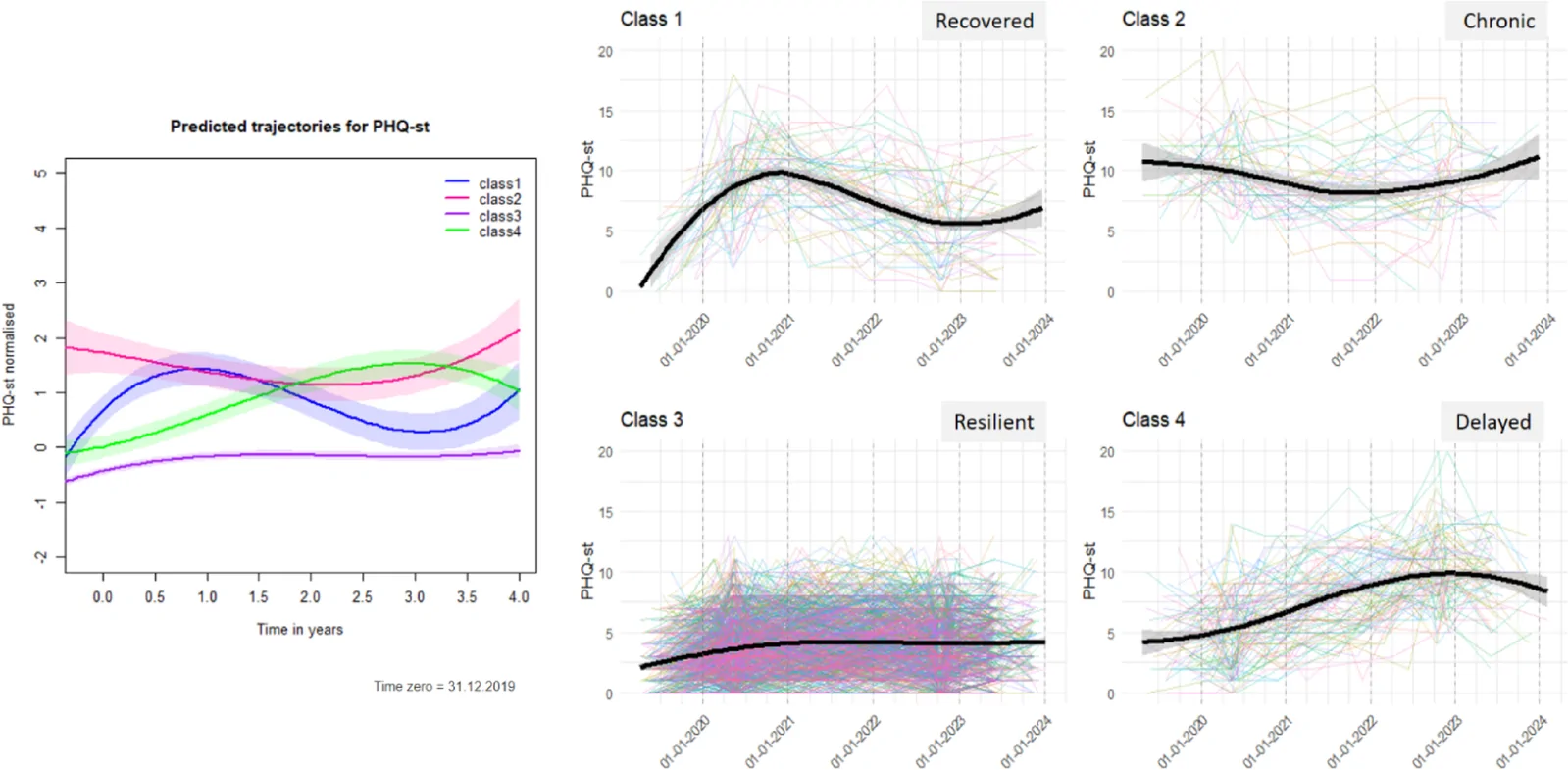
Predicted trajectories of PHQ-stress scores using LCMM analysis (on the left side) and spaguetti plots of individual trajectories by class with a LOESS smoothed trend line (on the right side), and with the respective class label

Based on the criteria described in the Methods section, we selected a four-class model (Fig. 2). The average posterior probabilities for class membership were 0.77, 0.80, 0.93, and 0.79 for Classes 1 to 4, respectively. Predicted trajectories of PHQ-st scores and individual PHQ-st trajectories by class are presented in Fig. 3.

Class 1 started with relatively low pre-pandemic stress levels and exhibited the steepest increase in the first year, reaching the highest point in the last months of 2020, followed by a decline that did not return to the pre-pandemic values by the end of the study period. This class included 52 (5.4%) participants, and can be labeled as the recovered class. Class 2 showed a trajectory with consistent higher stress levels, with a tendency to decrease between 2021 and 2022, but increasing again afterwards. This class comprised 38 (3.9%) participants, and was named as the chronic class. Class 3 presented the lowest pre-pandemic stress levels compared to the other classes, and remained in low to moderate levels overall, with a slight increase during the early phases of the pandemic that persisted throughout the observed period. This resilient class, was the biggest (81.7%, *n* = 789), had the highest proportion of men (48.3%, *n* = 379) and with the highest mean age (53 years, SD = 13). Class 4 began with relatively moderate levels of stress, but exhibited a peak later, in the last months of 2022. This class encompassed 87 (9.0%) of the participants, and was labeled as the delayed class.

### Multinomial and binomial logistic regression

The findings from multinomial logistic regression should be interpreted with caution due to the size of some classes compared to the resilient class – which was used as the reference class, because of its conceptual relevance and because it was the largest group. The binomial logistic regression model was performed aiming to improve precision, stability, and power, but with it class-specific information was reduced (Table 3  and SM).

Reporting lower life satisfaction at FU1, consistently increased the odds of belonging to all of the non-resilient trajectories when comparing to the resilient class. For other covariates, the estimates were not always consistent between the multinomial and binomial models. From the binomial analysis, being older, and being more agreeable was associated with membership in the resilient class. In the opposite direction, being employed at the beginning of the pandemic was associated with membership in non-resilient classes. Participants with higher baseline psychosocial stress (except recovered), and whose ever got diagnosed with depression (in multinomial only significant for the delayed class) were also more likely to follow non-resilient trajectories. Moreover, not having German as a mother tongue (a probable proxy of migration history) seemed to be associated with less resilient trajectories. Although the estimates should be interpreted with caution due to the small size of the chronic class, the observed effect was large (OR = 0.08, 95% CI: 0.01–0.71).

**Table 3:** Odds ratios and 95% confidence intervals from **multivariable multinomial and binomial logistic regressions** comparing membership in the resilient trajectory (reference class) with other classes associated with covariates from baseline, at FU1 and at CO1, n (total) = 956

Collection	PHQ-st trajectories’ classes	Multinomial			Binomial
		Class (…) vs. Reference class: Resilient (n 780; 81.6%)			
		Recovered (n 52; 5.4%)	Chronic (n 37; 3.9%)	Delayed (n 87; 9.0%)	Non-resilient (n 176; 18.4%)
		OR [95% CI]			
	Covariates				
Baseline	Female sex (Ref = Male sex)	1.08 [0.54, 2.16]	1.71 [0.66, 4.41]	0.85 [0.51, 1.44]	1.01 [0.66, 1.54]
	Age in January 2019	0.97 [0.94, 1.01]	0.98 [0.93, 1.03]	0.97 [0.94, 1.00]	0.97 [0.95, 0.99]
	German is a mother tongue: Yes (Ref = No)	0.22 [ 0.04, 1.21]	0.08 [ 0.01, 0.71]	0.62 [0.13, 3.09]	0.33 [0.11, 1.08]
	BIG 5 Agreeableness score	0.70 [0.51, 0.98]	0.80 [0.51, 1.24]	0.83 [0.64, 1.06]	0.77 [0.63, 0.95]
	BIG 5 Consciousness score	1.42 [0.97, 2.10]	1.04 [0.64, 1.70]	1.05 [0.80, 1.38]	1.16 [0.93, 1.46]
	BIG 5 Extraversion score	1.03 [0.81, 1.32]	1.09 [0.77, 1.54]	1.21 [0.99, 1.48]	1.13 [0.96, 1.32]
	BIG 5 Openness score	1.16 [0.88, 1.53]	1.24 [0.84, 1.83]	0.90 [0.73, 1.11]	1.01 [0.85, 1.19]
	BIG 5 Neuroticism score	1.42 [1.07, 1.88]	1.39 [0.94, 2.05]	0.96 [0.77, 1.19]	1.11 [0.94, 1.32]
	Childhood trauma score (CTS)	1.11 [0.98, 1.25]	1.04 [0.88, 1.22]	0.95 [0.85, 1.07]	1.01 [0.93, 1.10]
	Psychosocial stress (PHQ-st)	1.08 [0.96, 1.21]	1.44 [1.26, 1.65]	1.19 [1.09, 1.31]	1.20 [1.12, 1.29]
	International socio-economic index (ISEI)	1.00 [0.98, 1.02]	1.01 [0.98, 1.04]	1.01 [0.99, 1.02]	1,00 [0.99, 1.02]
FU1	Divorced: Yes (Ref = No)	0.99 [0.37, 2.69]	0.54 [0.11, 2.73]	1.97 [1.03, 3.8]	1.38 [0.77, 2.43]
	Life satisfaction (L-1)	0.74 [0.62, 0.87]	0.74 [0.59, 0.93]	0.83 [0.72, 0.95]	0.79 [0.71, 0.88]
	Health-related quality of life (SF36)	1.00 [0.58, 1.72]	1.87 [0.92, 3.83]	1.18 [0.77, 1.79]	1.20 [0.86, 1.68]
	Ever diagnosed with Depression: Yes (Ref = No)	1.23 [0.51, 2.97]	1.33 [0.44, 4.03]	2.61 [1.37, 4.96]	1.94 [1.13, 3.30]
	Ever diagnosed with Anxiety/Panic attack: Yes (Ref = No)	0.87 [0.28, 2.71]	1.03 [0.27, 3.95]	0.82 [0.35, 1.94]	0.85 [0.42, 1.68]
	BMI: ≥25 kg/m^2^ (Ref = < 25 kg/m^2^)	1.87 [0.93, 3.75]	2.00 [0.74, 5.40]	1.11 [0.65, 1.89]	1.37 [0.90, 2.10]
CO1	Employment: Yes (Ref = No)	3.19 [1.08, 9.45]	7.37 [1.78, 30.55]	1.33 [0.65, 2.71]	1.98 [1.10, 3.65]
	Household finances difficulties: Yes (Ref = No)	1.14 [0.46, 2.79]	2.09 [0.72, 6.01]	0.78 [0.34, 1.78]	1.13 [0.62, 2.00]
	Single parents: Yes (Ref = No)	1.70 [0.43, 6.79]	0.84 [0.12, 6.05]	1.30 [0.37, 4.56]	1.37 [0.50, 1.50]
	Living with partner and minor child(ren): Yes (Ref = No)	1.16 [0.53, 2.54]	1.18 [0.38, 3.61]	0.74 [0.39, 1.42]	0.91 [0.55, 1.50]
	Loneliness score	1.13 [0.92, 1.39]	1.30 [0.99, 1.71]	1.07 [0.90, 1.26]	1.11 [0.97, 1.26]
	Increased activities in the household: Yes (Ref = No)	2.82 [1.44, 5.50]	0.84 [0.31, 2.26]	1.06 [0.60, 1.85]	1.40 [0.91, 2.15]
	Decreased activities getting around: Yes (Ref = No)	1.48 [0.73, 2.97]	1.37 [0.50, 3.76]	0.50 [0.27, 0.92]	0.81 [0.52, 1.26]
	Decreased sport activities: Yes (Ref = No)	1.03 [0.52, 2.05]	0.56 [0.21, 1.54]	1.66 [1.00, 2.77]	1.24 [0.82, 1.87]
	Number of fears	1.14 [1.00, 1.30]	1.21 [1.01, 1.45]	0.99 [0.89, 1.10]	1.06 [0.97, 1.15]

Abbreviations: BMI, body mass index; CO1, 1^st^ COVID-9 questionnaire; FU1, 1^st^ follow-up examination; kg, kilogram; n, number of participants; m, meter; PHQ-st, the Patient Health Questionnaire - stress module;Scores ranges:

- Ascending order: Big-5:1 to 7; CTS: 5 to 25; number of fears at CO1: 0 to 8; ISEI: 10 to 89; L-1: 1 to 11; Loneliness: 3 to 9; PHQ-st: 0 to 20;

- Descending order: SF36-1 item: 1 (excellent) to 5 (poor)

Nagelkerke’s R^2^ values: multinomial = 0.37; binomial = 0.31

## Discussion

### Main findings and integration with previous literature

We identified four distinct psychosocial stress trajectories from pre- to post-pandemic, indicating heterogeneity in stress responses. These trajectories align with prior evidence showing heterogeneous mental distress responses to potential traumatic events [17, 33, 34]. Within this context, Bonanno’s framework [33] offers a useful lens, describing four typical trajectories after adversity: resilience (stable psychological well-being, despite exposure to adversity and temporary increases in distress), recovery (impairment followed by improvement), chronic dysfunction (persistent distress), and delayed dysfunction (later deterioration). In this framework, resilience predominates (55–65%), while recovery (15–25%), chronic (5–30%), and delayed (≤ 15%) trajectories are less frequent [33]. Because resilience is most common, population averages can mask subgroup patterns, and thus it is important to explore further whether heterogeneity might be present, using methods that allow this, such as grow mixture modelling [62].

Our data showed trajectories similar to the ones described in Bonanno’s framework, and were therefore labeled likewise. Most participants, around 82%, followed a resilient trajectory, while the rest of participants were more likely to belong to other trajectories, grouped as non-resilient. Early in the pandemic all classes showed an increase in stress levels, varying in different magnitude across classes, and a full recovery to pre-pandemic levels did not appear during the observed time.

Compared to the trajectories described by Bonanno, there is a partial divergence in the recovered class, which seems to exhibit a less pronounced recovery. This possibly reflects that Bonanno’s framework was mainly developed based on acute potentially traumatic events, while the pandemic represents a prolonged, multi-stressor context with many layers beyond infection itself and gradual onset, amplifying distress for some individuals and influencing post-traumatic adaptation in ways that differ from past crises [21, 33]. Containment measures disrupted work, household routines, and social interactions, while also limiting access to social and emotional support [63–67]. Moreover, while not everyone experienced additional adverse events (e.g., bereavement or illness), when such events occurred, their impact was potentially intensified by pandemic-related constraints, such as limited visits, support, and mourning rituals [68–71]. One may also link the delayed trajectory to other potential traumatic events, such as the Russian invasion of Ukraine in February 2022 [11, 72] and the subsequent energy crisis and inflation [9, 13]. Since the PHQ-st does not assess war-related stressors, a direct association is unlikely. However, these events may have attenuated recovery in stress levels. Other instruments, including measures more sensitive to war-related experiences, or broader aspects of mental health (e.g. anxiety or depression symptoms), might capture changes not detected in this study.

Studies on response to the COVID-19 pandemic that measured trajectories of mental health also have found similar trajectories; those most commonly investigated depressive symptoms, anxiety symptoms, and general mental distress, while stress was less common, or integrated in other mental-related scales. As expected, these studies identified resilience as the most common trajectory, constituting around two thirds of the samples, other trajectories included recovered (with approximately 13%), delayed distress (around 12%), and chronic distress (around 11%). Some studies also identified a moderate-stable distress trajectory [17]. Specifically on stress, we have identified two studies in Germany, which found three [39] and four [38] trajectories, using different instruments, the Perceived Stress scale [39] and the DASS-21 [38], respectively. In these studies, most participants showed resilience [38, 39].

Overall, these findings are consistent with the broader international literature, while extending it by examining psychosocial stress trajectories over a longer observation period and by contributing to the application of Bonanno’s framework in the context of prolonged, population-wide stressors such as pandemics.

### Factors associated with class membership

Overall, non-resilience trajectories of psychosocial stress during the pandemic seemed to be mainly associated with pre-existing psychological vulnerability, lower life satisfaction, younger age, and employment at the beginning of the pandemic. These findings are mostly consistent with previous research on mental health trajectories during the COVID-19 pandemic [17, 35, 36, 38, 39].

Individuals belonging to non-resilient classes reported more anxiety and depressive symptoms before the pandemic. Similar findings have been reported [73, 74], showing that pre-existing anxiety and depressive symptoms, predicted worse mental health during the pandemic, whereas fewer prior issues were associated with greater resilience [70, 75–78]. Having received a diagnosis of depression before the pandemic may have increased vulnerability to non-resilient trajectories, as previous depressive episodes can alter stress reactivity and lower the threshold for later deterioration under prolonged stress [79]. Additionally, pandemic-related disruptions in access to mental health care, including cancelled appointments, may have contributed to delayed worsening among individuals with pre-existing mental health conditions [80].

Several studies have found a positive association between age and resilience, as well as better psychological well-being, which is explained by greater life experience and coping resources [81, 82]. Higher life satisfaction is often associated with resilience, as it may reflect greater psychological resources, a more positive appraisal of life demands, and a higher tolerance for uncertainty, facilitating adaptability, as also reported during the pandemic [83–86].

In our study, being employed at the beginning of the pandemic was positively associated with membership in non-resilient trajectories, which diverges from previous research suggesting that employment may act as a protective factor for mental health [87]. One possible explanation is that not being employed in our sample does not necessarily reflect job loss or economic disadvantage, but may also include individuals who were already retired. Indeed, the resilient class in our study tended to be older, which may indicate that participants were at different life stages, potentially characterised by greater stability and less concerns, or the absence of work-related demands [88–91]. This suggests that employment status may reflect differences in life stage rather than a direct protective or risk factor. In addition, evidence from systematic reviews suggests that the pandemic extensively changed working conditions and introduced new occupational stressors, including remote work, increased workload, and job insecurity, contributing to higher levels of work-related stress among many workers [92–94].

The association between not having German language as a mother tongue with less resilient trajectories is consistent with evidence suggesting worse mental health among individuals with a migration history, which has been linked to socioeconomic disadvantage, language barriers, and reduced access to social support [95], and was particularly relevant during the pandemic [96–98].

In fact, the lack of social interactions and support may exacerbate the stress response. Kang et al. found that individuals with high loneliness are more susceptible to everyday stressors and tend to experience prolonged emotional reactions [99]. In our study, it was in a similar direction but not strong enough to draw firm conclusions; a similar pattern was observed for covariates related to behavioural changes. However, during the pandemic, physical distancing likely disrupted social interactions and potentially amplified stress responses [70, 100]. This way, the decrease in sports activities may have affected coping resources of some individuals, by affecting the role of physical activity in reducing stress and decreasing chances of social interactions and support [101–106].

Moreover, membership in non-resilient classes, particularly in the recovered class, was negatively associated with agreeableness, a personality trait linked to trust, cooperation, and empathy. Low agreeableness, characterized by scepticism, individualism, and less adaptability [107], may exacerbate stress vulnerability when routines, relationships, or coping mechanisms are disrupted, as during the pandemic. Such individuals report more stressors, and greater difficulty managing relationships and stress [108]. In our study, the associations related to relationships and family structures were also unclear.

Taken together, stress responses are shaped by a complex interplay of individual characteristics and contextual factors. Even under similar environmental conditions, individuals may react differently depending on sociodemographic background, personality traits, life experiences, prior trauma, and coping resources [109, 110]. However, recent evidence based on pandemic-related studies, suggests that neither single resilience factors nor their combined effects sufficiently predict who follows a resilience trajectory [17], a phenomenon referred to as the *resilience paradox* [111]. These findings suggest that resilience is better understood as a dynamic adaptive process and highlight the importance of studying mental distress in relation to stressor exposure and stress responses [17].

### Strengths and limitations

To our knowledge, no other studies in Germany have investigated heterogeneous psychosocial stress trajectories both before the pandemic and over a comparable duration. The major strength of this study lies in its contribution to the evidence on trajectories following adverse events, and to resilience literature. Our findings support the applicability of Bonanno’s framework to the context of pandemics.

Despite these strengths, several limitations should be acknowledged. Vulnerable groups, such as migrants, who tend to have a higher prevalence of stress and mental health problems [112, 113], are underrepresented in the NAKO study [114, 115]. Similarly, other known vulnerability factors in Germany, such as younger age and financial difficulties [8, 109], were less frequent in our predominantly old and financially comfortable sample. This may lead to an underestimation of the prevalence of more adverse stress trajectories typical of vulnerable groups, while the overall trajectory patterns are less likely to be affected.

Non-response or lost to follow-up bias, a common issue in longitudinal studies, may have been further aggravated by the inclusion criteria of our sample.

Another limitation concerns the measurement instrument (PHQ-st), which was not specifically developed for large-scale societal disruptions and may not capture context-specific stressors;, also its 3-point response scale may reduce sensitivity to subtle inter- and intra-individual changes.

While not a limitation per se, the number and form of latent classes identified through LCMM may vary depending on sample characteristics and measurement intervals. Thus, different trajectory patterns may have emerged under alternative study conditions.

## Conclusions

The identification of four stress trajectories reveals heterogeneity in population-level responses to the COVID-19 pandemic. Most participants showed resilience, and a small portion exhibited dysfunctional trajectories. However, none of the classes returned to initial levels, therefore it is important to keep tracking stress levels, for better understanding of potential lasting psychological impact.

These findings suggest that public health efforts to reduce chronic stress and support for individuals with psychosocial, health, or socio-demographic disadvantages, should be sustained beyond periods of crisis. Clinicians should identify individual vulnerabilities and stressors that might impact resilience when evaluating and supporting patients.

## Supplementary Information

Below is the link to the electronic supplementary material.


Supplementary Material 1


## Data Availability

The NAKO data that support the findings of this study are available from TransferHub (https://transfer.nako.de/transfer/index), but restrictions apply to the availability of these data, which were used under license for the current study and so are not publicly available. The data are, however, available upon request and with the permission of NAKO e.V.
