# Supplementary material for: Psychosocial stress from pre- to post-pandemic times: latent class mixed model analysis using data from a German cohort
Source: Soc Psychiatry Psychiatr Epidemiol. 2026 Apr 20;61(8):1565–78. doi: 10.1007/s00127-026-03092-6 (PMC13522046; doi:10.1007/s00127-026-03092-6)
Supplement: Supplementary file 1 [file 127_2026_3092_MOESM1_ESM.docx]

Supplementary material (SM)

|  | Not bothered | Bothered a little | Bothered a lot |
| --- | --- | --- | --- |
| 1. Worries about health | 0 | 1 | 2 |
| 2. Worries about weight or appearance | 0 | 1 | 2 |
| 3. Little or no sexual desire or pleasure during intercourse | 0 | 1 | 2 |
| 4. Difficulties with partner | 0 | 1 | 2 |
| 5. Stress from caring for children, parents, or other family members | 0 | 1 | 2 |
| 6. Stress at work or school | 0 | 1 | 2 |
| 7. Financial problems or worries | 0 | 1 | 2 |
| 8. Having no one to turn to with problems | 0 | 1 | 2 |
| 9. Something bad that happened recently | 0 | 1 | 2 |
| 10. Thinking about terrible events from the past or dreaming about them – e.g., the destruction of your home, a serious accident, physical violence, or a forced sexual act | 0 | 1 | 2 |

Figure 1 – English translation of the German version of the Patient Health Questionnaire - stress module (PHQ-st)

Figure 2 - Results from the BLRT analysis

| **Comparison** | **LR statistic** | **BLRT p-value** | **Bootstrap replications (B)** |
| --- | --- | --- | --- |
| 3 vs. 4 classes | 76.14 | 0.004 | 499 |

**Note:** Bootstrap Likelihood Ratio Test (BLRT) was computed comparing 3 vs 4 classes using a parametric bootstrap approach.

- McLachlan, G.J. (1987). On bootstrapping the likelihood ratio test statistic for the number of components in a normal mixture. Applied Statistics, 36(3), 318-324.

- Lo, Y., Mendell, N.R. & Rubin, D.B. (2001). Testing the number of components in a normal mixture. Biometrika, 88(3), 767-778.

- Nylund, K.L., Asparouhov, T. & Muthén, B.O. (2007). Deciding on the number of classes in latent class analysis and growth mixture modeling. Structural Equation Modeling, 14(4), 535-569.

**Complementary information on covariates**

| **Covariates** | **time point** |
| --- | --- |
| Sex | Baseline |
| Age | Baseline |
| German as one mother tongue | Baseline |
| Early childhood trauma (CTS) | Baseline |
| Personality traits (Big 5) | Baseline |
| international social-economic index of occupation status (ISEI) | Baseline |
| Monthly household net income | Baseline |
| Marital Status | Baseline + FU1 |
| Relationship Status | Baseline + FU1 |
| Household members | Baseline + FU1 |
| Housing structure | CO1 |
| Employment Status | Baseline + FU1+CO1 |
| Occupation | Baseline + FU1 |
| Education (ISCED97) | Baseline + FU1 |
| Anxiety symptoms (GAD-7), | Baseline + FU1+CO1 |
| Depressive symptoms (PHQ-9) | Baseline + FU1+CO1 |
| Health-related quality of life (SF36 - 1 item), | Baseline + FU1+CO1 |
| Diagnosed Anxiety/panic attack | Baseline + FU1 |
| Diagnosed Depression | Baseline + FU1 |
| Body mass index (BMI) | Baseline + FU1 |
| Life satisfaction (L-1) | Baseline + FU1+CO1 |
| Quality of sleep (PSQI) | Baseline |
| Loneliness | CO1 |
| Behavioural changes | CO1 |
| Household financial strain | CO1 |
| Number of fears | CO1 |

We considered month and year of birth, sex, mother tongue, early childhood trauma (assessed by the childhood trauma screener) (38) and personality traits (assessed by the big 5 inventory) (37) as time-invariant variables, and those were collected at baseline examination.

At both baseline and follow-up examination, participants were inquired about their marital status (single, married living together, married living apart, divorced, and widowed) and relationship status (living with a long-term-partner, having a long-term partner not living together or not having a long-term partner). The assessments also included questions on employment status (employed, unemployed, non-working person), and current occupation. For occupation, categories included student, pensioner, permanently unable to work, housewife/househusband or other. Educational background was estimated based on the highest level of formal education and the vocational training, classified according to the International Standard Classification of Education (ISCED 2011) (39). From baseline, a variable indicating the international social-economic index of occupation status (ISEI) (40, 41) was available and we included it in our analysis. As well as for the variable Social Network Index, which derives from marital status, contact with family and friends, and group memberships, and scores from 1 to 4, with 1 indicating social isolation^(1,2).^

Housing situation was also assessed through questions on number of household members aged fourteen years or older, number of children under that age, and monthly net household income. These questions were formulated differently at baseline, FU1 and CO1: all were available for baseline, for FU1 only the number of member, and for CO1 the possible answers were about the household structure (people living alone, couples without children in the household, couples with minor child(ren) in the household, couples with adult child(ren) in the household, single parents, or other multi-person households).

Anxiety symptoms were measured by the Generalised Anxiety Disorder-7 (GAD-7) screener, which is a seven-item scale measuring symptom severity on a scale from ‘Not at all’ (0) to ‘Nearly every day’ (3), obtaining a score ranging from 0 to 21, with higher scores indicating higher anxiety (42). Depressive symptoms were assessed by the Patient Health Questionnaire-9 (PHQ-9), using the same 0–3 scale as the GAD-7 but for nine items, for a total score ranging from 0 to 27 (43).

Health-related quality of life was assessed using a single item from the 36-Item Short Form Survey (SF-36), where responses ranged from 1 (excellent) to 5 (poor), with intermediate options of 2 (very good), 3 (good), and 4 (fair) (44).

Participants also reported previous diagnoses of mental disorders by a physician (depression and anxiety or panic attacks) at baseline and FU1. For the first follow-up, we combined the variables from those two examinations and reported as an ‘ever diagnosed’ until then.

Height and weight were recorded at the study centre, and the body mass index (BMI) was determined by dividing the weight in kilograms by the height in meters squared, and then categorized according to WHO criteria (45).

One question about life satisfaction was also included, the general life satisfaction short scale (L-1), ‘How satisfied are you currently, all in all, with your life?’, which is a 11-point rating scale from 1 ‘not at all satisfied’ to 11 ‘completely satisfied’ (46).

We included one variable from the Pittsburgh Sleep Quality Index (PSQI): the item assessing subjective sleep quality at baseline (47).

In the first corona questionnaire (CO1), new questions were introduced regarding the pandemic and its impact, including changes in behaviours and broader effects on participants' lives. In CO1, participants responded about their household’s financial situation in terms of their ability to meet expenses. Responses were categorized as: ‘easy’, ‘reasonably easy’, ‘with some difficulties’, and ‘with great difficulty’.

Regarding changes in activities, questions focused on various aspects of daily life, such as household activities, seated activities, mobility (getting around), sports, and leisure activities. Participants were asked to rate these activities on a scale from: ‘much less than before’, ‘slightly less than before’, ‘remained the same’, ‘a little more than before’, to ‘much more than before’.

We included the loneliness score, measured by a 3 items scale about companionship missing, being left out, socially isolated, where 1 means ‘rarely or never’, 2 ‘sometimes’, and 3 ‘often’, leading to a score from 3 to 9, higher scores indicating greater loneliness (48). The sum of fears reported in CO 1 also considered in the analysis, ranging from 0 to 8, fears included (road traffic accident, natural disaster, corona-virus infection, cancer, stroke, heart attack, diabetes mellitus, hereditary disease).

We transformed most of the categorical variables into 2 categories variables to address the reduced number of participants in some categories and to facilitate interpretation.

Rather than building a model using only the most recent covariates, we chose to test models progressively: first with baseline variables alone, then adding FU1 and CO1 assessments. This stepwise approach lets us first assess associations using baseline covariates, then examine how adding FU1 and CO1 measures changes the associations, while preserving temporal clarity.

1. Berkman LF, Syme SL. Social networks, host resistance, and mortality: a nine-year follow-up study of Alameda County residents. American journal of epidemiology. 1979;109(2):186-204.

2. Vonneilich N, Becher H, Bohn B, Brandes B, Castell S, Deckert A, et al. Associations of Migration, Socioeconomic Position and Social Relations With Depressive Symptoms – Analyses of the German National Cohort Baseline Data. International Journal of Public Health. 2023;Volume 68 - 2023.

Table 1 - Frequencies for missing values before and after imputation for the covariates used in the final multinomial logistic model

| Collection |  | Before imputation | | After imputation | |
| --- | --- | --- | --- | --- | --- |
|  |  | Missing values | | Missing values | |
|  | **Covariates** | Counts (n) | Percentage (%) | Counts (n) | Percentage (%) |
| Baseline | Sex | 0 | 0,0 | 0 | 0,0 |
|  | Age (birthday date) | 1 | 0,1 | 0 | 0,0 |
|  | German is a mother tongue | 0 | 0,0 | 0 | 0,0 |
|  | BIG 5 Agreeableness score | 10 | 1,0 | 0 | 0,0 |
|  | BIG 5 Consciousness score | 11 | 1,1 | 0 | 0,0 |
|  | BIG 5 Extraversion score | 12 | 1,2 | 0 | 0,0 |
|  | BIG 5 Openness score | 13 | 1,4 | 0 | 0,0 |
|  | BIG 5 Neuroticism score | 14 | 1,5 | 0 | 0,0 |
|  | Childhood trauma score (CTS) | 65 | 6,8 | 0 | 0,0 |
|  | Psychosocial stress (PHQ-st) | 16 | 1,7 | 0 | 0,0 |
|  | International socio-economic index (ISEI) | 55 | 5,7 | 0 | 0,0 |
| FU1 | Divorced | 0 | 0,0 | 0 | 0,0 |
|  | Life satisfaction (L-1) | 1 | 0,1 | 0 | 0,0 |
|  | Health-related quality of life (SF36) | 0 | 0,0 | 0 | 0,0 |
|  | Ever diagnosed with Depression | 1 | 0,1 | 0 | 0,0 |
|  | Ever diagnosed with Anxiety/Panic attack | 1 | 0,1 | 0 | 0,0 |
|  | BMI | 4 | 0,4 | 0 | 0,0 |
| CO1 | Employment | 57 | 5,9 | 5 | 0,5 |
|  | Household finances difficulties | 47 | 4,9 | 4 | 0,4 |
|  | Single parents | 46 | 4,8 | 0 | 0,0 |
|  | Living with partner and minor child(ren) | 46 | 4,8 | 0 | 0,0 |
|  | Loneliness score | 48 | 5,0 | 0 | 0,0 |
|  | Increased activities in the household | 49 | 5,1 | 4 | 0,4 |
|  | Decreased activities getting around | 49 | 5,1 | 4 | 0,4 |
|  | Decreased sport activities | 49 | 5,1 | 4 | 0,4 |
|  | Number of fears | 70 | 7,3 | 0 | 0,0 |

Table 2 - Frequencies of baseline covariates for included sample and non-responders (baseline participants) to follow-up examinations

| **Baseline Covariates** | **Included (n = 961)** | **Non-responders (n = 9178)** |
| --- | --- | --- |
| **Female sex (%)** | 53.28% | 51.65% |
| Age in January 2019, mean (SD) | 52.41 (12.55) | 52.03 (12.76) |
| **German is one mother tongue (%)** | 97.81% | 96.38% |
| International socio-economic index (ISEI) , mean (SD) | 53.53 (14.95) | 47.48 (15.60) |
| **Education in years**, mean (SD) | 16.20 (2.06) | 15.46 (2.14) |
| Psychosocial stress (PHQ-st), mean (SD) | 3.49 (2.97) | 3.45 (3.04) |
| Depressive symptoms (PHQ-9 score), mean (SD) | 3.47 (3.33) | 3.73 (3.61) |
| Anxiety symptoms (GAD-7 score), mean (SD) | 3.07 (3.11) | 2.99 (3.11) |
| Life satisfaction (L-1), mean (SD) | 8.87 (1.74) | 8.73 (1.91) |
| Health-related quality of life (SF36 - 1 item), mean (SD) | 2.68 (0.66) | 2.82 (0.67) |

*Table 3a – Mean and Standard Deviation of Participant Characteristics at Baseline not included in the paper, by PHQ-Stress Trajectory Class (Numeric Covariates)*

| Collection time | Participants  Covariates |  | PHQ-st trajectories’ classes | | | |
| --- | --- | --- | --- | --- | --- | --- |
|  |  | All sample, n (%) | Recovered,  n (%) | Chronic,  n (%) | Resilient,  n (%) | Delayed,  n (%) |
|  |  | 961 (100) | 52 (5.4%) | 38 (3.9%) | 789 (81.6%) | 87 (9.0%) |
| Baseline | Age in January 2019^a^, mean (SD) | 52.40 (12.54) | 46.19 (10.16) | 47.71 (10.07) | 53.38 (12.62) | 49.34 (12.23) |
|  | Health-related quality of life (SF36 - 1 item), mean (SD) | 2.68 (0.66) | 2.75 (0.59) | 3.11 (0.76) | 2.64 (0.65) | 2.82 (0.69) |
|  | Life satisfaction (L-1), mean (SD) | 8.87 (1.73) | 8.58 (1.61) | 7.34 (2.26) | 9.04 (1.64) | 8.26 (1.88) |
|  | Anxiety symptoms (GAD-7 score), mean (SD) | 3.04 (3.10) | 4.67 (4.37) | 7.71 (4.95) | 2.60 (2.57) | 4.00 (3.40) |
|  | Depressive symptoms (PHQ-9 score), mean (SD) | 3.46 (3.33) | 4.54 (3.97) | 8.26 (5.48) | 2.99 (2.77) | 4.91 (4.08) |
|  | International socio-economic index (ISEI) , mean (SD) | 53.08 (15.05) | 51.46 (15.35) | 48.53 (14.68) | 53.52 (15.01) | 52.10 (15.21) |
| Scores ranges:   - Ascending order: Big-5 traits: 1 to 7; CTS: 5 to 25; ISEI: 10 to 89; GAD-7: 0 to 21; number of fears: 0 to 8; L-1: 1 to 11; Loneliness: 3 to 9; PHQ-9: 0 to 27; PHQ-st: 0 to 20; - Descending order: SF36-1 item: 1 (excellent) to 5 (poor)   ^a^ calculated from month and year of birth collected at baseline | | | | | | |

Table 3b – Participant Characteristics not included in the paper, by PHQ-Stress Trajectory Class (Frequencies and Percentages for Categorical Variables)

| PHQ-st trajectories’ classes | All sample, n (%) | Recovered, n (%) | Chronic, n (%) | Resilient, n (%) | Delayed, n (%) |
| --- | --- | --- | --- | --- | --- |
| Expected participants, n (%) | 961 (100) | 52 (5.4) | 38 (3.9) | 789 (81.6) | 87 (9.0) |
| German language proficiency | | | | | |
| German as mother tongue | 934 (97.2) | 47 (90.4) | 35 (92.1) | 767 (97.8) | 85 (97.7) |
| German and other language(s) as mother tongue | 6 (0.6) | 3 (5.8) | 0 (0) | 3 (0.4) | 0 (0) |
| Mother tongue not German, very good knowledge of German | 16 (1.7) | 1 (1.9) | 1 (2.6) | 12 (1.5) | 2 (2.3) |
| Mother tongue not German, good knowledge of German | 2 (0.2) | 0 (0) | 1 (2.6) | 1 (0.1) | 0 (0) |
| Mother tongue not German, mediocre German language skills | 3 (0.3) | 1 (1.9) | 1 (2.6) | 1 (0.1) | 0 (0) |
| Mother tongue not German, poor German language skills | 0 (0) | 0 (0) | 0 (0) | 0 (0) | 0 (0) |
| Mother tongue not German, very poor knowledge of German | 0 (0) | 0 (0) | 0 (0) | 0 (0) | 0 (0) |
| Ever diagnosed with Depression until baseline | | | | | |
| no, n (%) | 841 (87.5) | 42 (80.8) | 28 (73.7) | 705 (89.9) | 66 (75.9) |
| yes, n (%) | 120 (12.5) | 10 (19.2) | 10 (26.3) | 79 (10.1) | 21 (10.1) |
| Ever diagnosed with Anxiety/Panic attack until baseline | | | | | |
| no, n (%) | 894 (93) | 46 (88.5) | 34 (89.5) | 733 (93.5) | 81 (93.1) |
| yes, n (%) | 67 (7) | 6 (11.5) | 4 (10.5) | 51 (6.5) | 6 (6.9) |
| Body Mass Index at baseline | | | | | |
| under-/normal-weight (< 25kg/m^2^), n (%) | 453 (47.1) | 23 (4.2) | 14 (36.8) | 372 (47.4) | 44 (50.6) |
| overweight/obesity (≥25kg/m^2^), n (%) | 508 (52.9) | 29 (55.8) | 24 (63.2) | 412 (52.6) | 43 (49.4) |

Table 4 - Participant Characteristics not included in the paper, by PHQ-Stress Trajectory Class (Frequencies and Percentages for Categorical Variables)

| Collection |  | All sample, n (%) | PHQ-st trajectories’ classes | | | |  |
| --- | --- | --- | --- | --- | --- | --- | --- |
|  |  |  | Recovered, n (%) | Chronic, n (%) | Resilient, n (%) | Delayed, n (%) |  |
|  | Expected number of participants | 961 (100) | 52 (5.4) | 38 (3.9) | 789 (81.6) | 87 (9.0%) |  |
| COVID-19 first questionnaire | Impact of COVID-19 pandemic on sports activity (e.g. jogging, athletic cycling, weight training) ^(4 missing values)^ | | | | | | |
|  | Much less than before, n (%) | 221 (23.1) | 14 (26.9) | 8 (21.6) | 175 (22.4) | 24 (27.6) |  |
|  | Slightly less than before, n (%) | 214 (22.4) | 12 (23.1) | 6 (16.2) | 174 (22.3) | 22 (25.3) |  |
|  | Remained the same, n (%) | 379 (39.6) | 17 (32.7) | 14 (45.9) | 316 (40.5) | 29 (33.3) |  |
|  | A little more than before, n (%) | 114 (11.9) | 8 (15.4) | 5 (13.5) | 92 (11.8) | 9 (10.3) |  |
|  | Much more than before, n (%) | 29 (3.0) | 1 (1.9) | 1 (2.7) | 24 (3.1) | 3 (3.4) |  |
|  | Impact of COVID-19 pandemic on activity in the household (e.g. washing the dishes, cleaning, cooking) ^(4 missing values)^ | | | | | |  |
|  | Much less than before, n (%) | 5 (0.5) | 0 (0) | 0 (0) | 4 (0.5) | 1 (1.1) |  |
|  | Slightly less than before, n (%) | 28 (2.9) | 3 (5.8) | 2 (5.4) | 20 (2.6) | 3 (3.4) |  |
|  | Remained the same, n (%) | 688 (72.0) | 22 (42.3) | 23 (62.2) | 583 (74.6) | 60 (69.0) |  |
|  | A little more than before, n (%) | 189 (19.8) | 23 (44.2) | 8 (21.6) | 141 (18.1) | 17 (19.5) |  |
|  | Much more than before, n (%) | 47 (4.9) | 4 (7.7) | 4 (10.8) | 33 (4.2) | 6 (6.9) |  |
|  | Impact of COVID-19 pandemic on activity for getting around (e.g. walking, cycling to work/shopping) ^(4 missing values)^ | | | | | |  |
|  | Much less than before, n (%) | 87 (9.1) | 7 (13.5) | 3 (8.1) | 72 (9.2) | 5 (5.7) |  |
|  | Slightly less than before, n (%) | 177 (18.4) | 15 (28.8) | 11 (29.7) | 138 (17.7) | 13 (14.9) |  |
|  | Remained the same, n (%) | 534 (55.6) | 18 (34.6) | 15 (40.5) | 445 (57) | 56 (64.4) |  |
|  | A little more than before, n (%) | 136 (14.2) | 9 (17.3) | 6 (16.2) | 108 (13.8) | 13 (14.9) |  |
|  | Much more than before, n (%) | 23 (2.4) | 3 (5.8) | 2 (5.4) | 18 (2.3) | 0 (0) |  |

Table 5 – Odds ratios and 95% confidence intervals from **multivariable multinomial and binomial logistic regressions** comparing membership in the resilient class (reference class) with other classes associated with covariates from baseline, n (total) = 960

|  |  | Multinomial | | | Binomial |
| --- | --- | --- | --- | --- | --- |
|  |  | Class (…) vs Reference class: Resilient (n 780; 81.6%) | | | |
| Collection time | PHQ-st trajectories’ classes  Covariates | Recovered  (n 52; 5.4%) | Chronic  (n 37; 3.9%) | Delayed  (n 87; 9.0%) | Non-resilient  (n 176; 18.4%) |
|  |  | OR [95% CI] | | | |
| Baseline | Female sex (ref = Male sex) | 1.29 [0.66, 2.50} | 1.73 [0.73, 4.08] | 0.93 [0.56, 1.55] | 1.13 [0.76, 1.69] |
|  | Age in January 2019 | 0.95 [0.92, 0.98] | 0.97 [0.93, 1.01] | 0.96 [0.94, 0.99] | 0.96 [0.94, 0.98] |
|  | German is a mother tongue: Yes (Ref = No) | 0.29 [ 0.06, 1.49] | 0.07 [ 0.01, 0.43] | 0.74 [0.15, 3.65] | 0.35 [0.13, 1.01] |
|  | BIG 5 Agreeableness score | 0.71 [0.52, 0.96] | 0.70 [0.47, 1.05] | 0.82 [0.64, 1.04] | 0.77 [0.64, 0.94] |
|  | BIG 5 Consciousness score | 1.51 [1.04, 2.19] | 0.95 [0.61, 1.47] | 1.06 [0.81, 1.38] | 1.16 [0.94, 1.44] |
|  | BIG 5 Extraversion score | 0.97 [0.76, 1.24] | 1.04 [0.75, 1.46] | 1.13 [0.93, 1.38] | 1.07 [0.92, 1.23] |
|  | BIG 5 Openness score | 1.14 [0.87, 1.48] | 1.28 [0.89, 1.84] | 0.89 [0.73, 1.10] | 1.00 [0.85, 1.18] |
|  | BIG 5 Neuroticism score | 1.41 [1.07, 1.86] | 1.39 [0.98, 1.97] | 0.97 [0.78, 1.19] | 1.13 [0.96, 1.33] |
|  | Childhood trauma score (CTS) | 1.12 [1.00, 1.26] | 1.08 [0.93, 1.25] | 0.98 [0.88, 1.10] | 1.04 [0.96, 1.13] |
|  | Psychosocial stress (PHQ-st) | 1.16 [1.04, 1.31] | 1.49 [1.30, 1.70] | 1.19 [1.08, 1.31] | 1.21 [1.13, 1.30] |
|  | International socio-economic index (ISEI) | 1.00 [0.98, 1.03] | 1.00 [0.97, 1.03] | 1.00 [0.99, 1.02] | 1.00 [0.99, 1.02] |
|  | Social network index | 0.87 [0.55, 1.37] | 1.46 [0.79, 2.67] | 1.03 [0.73, 1.44] | 1.03 [0.78, 1.34] |
|  | Divorced: Yes (Ref = No) | 1.34 [0.50, 3.55] | 0.39 [0.07, 2.30] | 2.46 [1.29, 4.67] | 1.77 [1.00, 3.06] |
|  | Children under 14y in the household | 1.83 [0.94, 3.55] | 1.71 [0.71, 4.11] | 0.78 [0.43, 1.40] | 1,14 [0.74, 1.75] |
|  | Employed: Yes (Ref = No) | 2.38 [0.75, 7.54] | 3.59 [0.81, 15.83] | 1.29 [0.64, 2.60] | 1.68 [0.94, 3.10] |
|  | Life satisfaction (L-1) | 1.05 [0.85, 1.29] | 0.90 [0.71, 1.15] | 0.92 [0.80, 1.06] | 0.95 [0.84, 1.07] |
|  | Health-related quality of life (SF36) | 1.34 [0.79, 2.26] | 1.98 [1.03, 3.82] | 1.42 [0.94, 2.14] | 1.46 [1.06, 2.03] |
|  | Ever diagnosed with Depression: Yes (Ref = No) | 0.92 [0.35, 2.40] | 0.75 [0.24, 2.40] | 2.31 [1.19, 4.49] | 1.53 [0.87, 2.66] |
|  | Ever diagnosed with Anxiety/Panic attack: Yes (Ref = No) | 0.80 [0.23, 2.71] | 0.36 [0.07, 1.81] | 0.39 [0.14, 1.12] | 0.48 [0.21, 1.06] |
|  | BMI: ≥25kg/m^2^ (Ref = <25kg/m^2^) | 1.75 [0.90, 3.38] | 1.67 [0.68, 4.05] | 0.87 [0.52, 1.45] | 1.15 [0.77, 1.72] |
| Abbreviations: BMI, body mass index; kg, kilogram; n, number of participants; m, meter; PHQ-st, the Patient Health Questionnaire - stress module;  Scores ranges:   - Ascending order: Big-5:1 to 7; CTS: 5 to 25; number of fears at CO1: 0 to 8; ISEI: 10 to 89; L-1: 1 to 11; PHQ-st: 0 to 20; - Descending order: SF36-1 item: 1 (excellent) to 5 (poor)   Nagelkerke’s R^2^ values: multinomial = 0.30; binomial = 0.26 | | | | | |

Table 6 – Odds ratios and 95% confidence intervals from **multivariable multinomial and binomial logistic regressions** comparing membership in the resilient class (reference class) with other classes associated with covariates from baseline and FU1 , n (total) = 925

|  |  | Multinomial | | | Binomial |
| --- | --- | --- | --- | --- | --- |
|  |  | Class label vs Reference class: Resilient (n 780; 81.6%) | | | |
| Collection time | PHQ-st trajectories’ classes  Covariates | Recovered  (n 52; 5.4%) | Chronic  (n 37; 3.9%) | Delayed  (n 87; 9.0%) | Non-resilient  (n 176; 18.4%) |
|  |  | OR [95% CI] | | | |
| Baseline | Female sex (ref = Male sex) | 1.28 [0.65, 2.53] | 1.72 [0.69, 4.27] | 0.83 [0.50, 1.39] | 1.03 [0.68, 1.56] |
|  | Age in January 2019 | 0.97 [0.93, 1.00] | 0.98 [0.93, 1.02] | 0.97 [0.95, 1.00] | 0.97 [0.95, 0.99] |
|  | German is a mother tongue: Yes (Ref = No) | 0.51 [0.06, 4.48] | 0.12 [ 0.01, 1.24] | 0.66 [0.13, 3.22] | 0.45 [0.14, 1.64] |
|  | BIG 5 Agreeableness score | 0.72 [0.53, 0.98] | 0.74 [0.47, 1.15] | 0.87 [0.67, 1.12] | 0.80 [0.65, 0.98] |
|  | BIG 5 Consciousness score | 1.53 [1.05, 2.24] | 1.02 [0.64, 1.62] | 1.08 [0.82, 1.43] | 1.20 [0.97, 1.50] |
|  | BIG 5 Extraversion score | 1.05 [0.82, 1.34] | 1.08 [0.76, 1.53] | 1.15 [0.94, 1.40] | 1.10 [0.94, 1.29] |
|  | BIG 5 Openness score | 1.16 [0.89, 1.52] | 1.19 [0.81, 1.74] | 0.91 [0.74, 1.11] | 1.01 [0.86, 1.19] |
|  | BIG 5 Neuroticism score | 1.45 [1.09, 1.91] | 1.57 [1.06, 2.33] | 0.98 [0.79, 1.22] | 1.16 [0.98, 1.37] |
|  | Childhood trauma score (CTS) | 1.10 [0.98, 1.24] | 1.07 [0.91, 1.25] | 0.96 [0.85, 1.08] | 1.02 [0.94, 1.11] |
|  | Psychosocial stress (PHQ-st) | 1.12 [1.00, 1.26] | 1.46 [1.27, 1.67] | 1.19 [1.09, 1.30] | 1.21 [1.13, 1.30] |
|  | International socio-economic index (ISEI) | 1.00 [0.98, 1.03] | 1.00 [0.98, 1.05] | 1.00 [0.99, 1.02] | 1.00 [0.99, 1.02] |
| First follow-up examination | Divorced: Yes (Ref = No) | 1.11 [0.42, 2.96] | 0.53 [0.11, 2.55] | 1.90 [0.99, 3.65] | 1.44 [0.80, 2.53] |
|  | Children under 14y in the household | 1.34 [0.63, 2.86] | 0.99 [0.33, 2.97] | 0.66 [0.34, 1.28] | 0.88 [0.53, 1.43] |
|  | Employed: Yes (Ref = No) | 3.36 [1.10, 10.26] | 8.20 [1.78, 37.80] | 1.86 [0.91, 3.81] | 2.46 [1.36, 4.60] |
|  | Life satisfaction (L-1) | 0.75 [0.63, 0.90] | 0.69 [0.55, 0.87] | 0.84 [0.73, 0.96] | 0.79 [0.71, 0.89] |
|  | Health-related quality of life (SF36) | 1.02 [0.59, 1.74] | 1.58 [0.76, 3.28] | 1.04 [0.68, 1.60] | 1.11 [0.79, 1.56] |
|  | Ever diagnosed with Depression: Yes (Ref = No) | 1.15 [0.46, 2.86] | 1.40 [0.47, 4.16] | 2.55 [1.32, 4.95] | 1.90 [1.09, 3.27] |
|  | Ever diagnosed with Anxiety/Panic attack: Yes (Ref = No) | 1.01 [0.32, 3.14] | 1.01 [0.26, 3.93] | 0.80 [0.33, 1.92] | 0.88 [0.43, 1.74] |
|  | BMI: ≥25kg/m^2^ (Ref = <25kg/m^2^) | 1.78 [0.90, 3.54] | 2.05 [0.75, 5.55] | 1.14 [0.67, 1.93] | 1.38 [0.90, 2.11] |
| Abbreviations: BMI, body mass index; FU1, 1^st^ follow-up examination; kg, kilogram; n, number of participants; m, meter; PHQ-st, the Patient Health Questionnaire - stress module;  Scores ranges:   - Ascending order: Big-5:1 to 7; CTS: 5 to 25; ISEI: 10 to 89; L-1: 1 to 11; PHQ-st: 0 to 20; - Descending order: SF36-1 item: 1 (excellent) to 5 (poor)   Nagelkerke’s R^2^ values: multinomial = 0.32; binomial = 0.27 | | | | | |
